# Deep Learning Methods for Speed Estimation of Bipedal Motion from Wearable IMU Sensors

**DOI:** 10.3390/s22103865

**Published:** 2022-05-19

**Authors:** Josef Justa, Václav Šmídl, Aleš Hamáček

**Affiliations:** 1 Department of Measurement and Technology, Faculty of Electrical Engineering, University of West Bohemia, 30100 Pilsen, Czech Republic; hamacek@fel.zcu.cz; 2 Reseach and Innovation Center, Faculty of Electrical Engineering, University of West Bohemia, 30100 Pilsen, Czech Republic; vsmidl@rice.zcu.cz

**Keywords:** motion speed estimation, inertial measurement unit, deep learning, walking speed, autoencoder architecture

## Abstract

The estimation of the speed of human motion from wearable IMU sensors is required in applications such as pedestrian dead reckoning. In this paper, we test deep learning methods for the prediction of the motion speed from raw readings of a low-cost IMU sensor. Each subject was observed using three sensors at the shoe, shin, and thigh. We show that existing general-purpose architectures outperform classical feature-based approaches and propose a novel architecture tailored for this task. The proposed architecture is based on a semi-supervised variational auto-encoder structure with innovated decoder in the form of a dense layer with a sinusoidal activation function. The proposed architecture achieved the lowest average error on the test data. Analysis of sensor placement reveals that the best location for the sensor is the shoe. Significant accuracy gain was observed when all three sensors were available. All data acquired in this experiment and the code of the estimation methods are available for download.

## 1. Introduction

We are concerned with the problem of estimating the locomotion speed of an individual for the purpose of inertial indoor navigation. Specifically, our research aims at investigating indoor navigation in special situations where the traditional means of location such as GPS or infrastructure-based (WiFi) signals are not available. A typical example is an emergency situation resolution, e.g., locating a firefighter in a hostile unknown environment. Due to unreliable infrastructure, it is impossible to use vision-aided inertial systems (VINS) and magnetic or thermal fingerprint systems; thus, the Pedestrian Dead Reckoning (PDR) is the most reliable source of information about the location of the tracked person.

We are concerned with analyzing data from the inertial motion unit (IMU) sensors due to their robustness in hostile environments. The IMUs are assumed to be attached to the human body by an elastic band to ensure close synchronization of their movement with the body. This is not ensured in approaches using IMU in mobile phones, where synchronization is weaker. Due to the chosen application area, we aim to estimate a wide range of speeds for bipedal motion, i.e., walking as well as running. It is a narrower space than the general gait speed estimation [1], but it is wider than walking speed estimation [2] since we also include running.

Many classical PDR methods use manually designed features [1,2,3], often without detailed specification, which prevents their replication. One of the most popular features is the zero-velocity update (ZUPT) [4], which has been extensively used [5,6,7]. Detection of the feature itself may be a complicated problem requiring adaptive approaches [8]. The application of the approach to indoor navigation often requires a combination of multiple methods [9].

The limit of feature-based methods is poor generalization and difficult extension to more demanding scenarios. For example, the significance of the ZUPT feature decreases with the increasing speed of motion. Feature tuning or even the design of new features would be required to improve estimation in running, stairs walking, or ladder climbing, which are common in emergency rescue situations. Therefore, we do not aim to improve feature-based method but to investigate techniques that can learn to predict the speed from the raw data. The most active area of research where features are “discovered” automatically is deep learning [10], which is typically based on neural networks with many layers. The neural networks have been used for gait speed estimation early on [11], yet only in its simple form, which does not have the benefits of deep learning. Deep learning has been extensively applied to estimate human motion from videos [12], smartphones [13,14,15]. While the recognition of human speed from a smartphone is sufficient for low-risk applications, it is insufficient for the considered domain of emergency situation resolution. Therefore, we focus not only on the estimation of the speed from fixed mounted IMU sensors but also on the evaluation of the most suitable number and position of these sensors. The application of deep learning to locomotion speed detection from IMUs is much scarcer and is often based on simple network architectures. For example, Qian et al. [16] uses a feed-forward network with LSTM layers. In a parallel study, residual networks with BiLSTM architecture have been used to classify human activity [17].

The aim of our research is to evaluate modern general-purpose deep architecture that has been developed for modeling time-series. Specifically, we will investigate modern state-of-the-art methods such as the InceptionTime architecture that uses convolution layers with a bottleneck mechanism [18], the perceiver architecture [19] that uses the attention mechanism. Another successful approach that we will investigate is the semi-supervised variational autoencoder architecture. We test state-of-the-art architecture based on the composition of convolutional pre-processing layers and post-processing of LSTM [20,21] and extend it to autoencoders as suggested in [22] since it has been reported to outperform pure convolutional and recurrent networks.

The general architectures mentioned above do not use any specific information about the nature of the signal. Based on the character of the recorded signal, we propose a new architecture using networks with periodic activation functions. This architecture will be used as decoder in the semi-supervised approach. All architectures will be evaluated on data from eight subjects that were recorded for this study.

Our contributions can be summarized as follows:

1. We measured IMU data on eight human subjects while walking and running together with a reference speed recorded by a monowheel and provided those data as publicly. We also provide all codes to run the proposed methods to make replication of our results as convenient as possible.
2. We test existing architectures of deep learning for the task of predicting the motion speed from the IMU data. We show the benefits of approaches based on auto-encoder topology. Moreover, we propose a novel decoder architecture that achieves the best results on our datasets. The architecture is motivated by the nature of the IMU signal.
3. We provide sensitivity studies of the methods with respect to: (i) the subjects (via leave one out cross-validation), (ii) the number of IMU sensors on the body and their location, and (iii) availability of additional knowledge such as the length of the leg. We observed that these details are more important than the architecture of the neural network.

The work is structured as follows. A literature survey highlighting the most related approaches is provided in Section 2. Description of the data, problem formulation, and the main computational methods, including the proposed new architecture, are described in Section 3. All methods are evaluated on the recorded data in Section 4, where we show the superiority of the proposed architecture. The conclusion is presented in Section 6.

## 2. Related Work

We now briefly review the details of publications that we find the closest to our approach, as summarized in Table 1. From the range of classical feature-based methods, we select those related to neural networks.

### 2.1. Feature-Based Approaches

A large number of methods based on classical feature-based approaches are available; see summary in Table 1. Since the approach is sensitive to the tuning of the feature detection, many papers focus on improving the feature extraction, e.g., [7,23]. However, since our primary aim is to learn from the raw data, we will consider feature-based methods as a baseline for comparison. From a wide range of available methods, we analyze the performance of methods mentioned in survey [24], including extensive parameter tuning. Moreover, the default sensor placement in these approaches is almost always determined to be the foot attachment [4], with few exception such as [25]. We will study the sensitivity of this choice to estimation precision in the experimental section.

### 2.2. Neural Networks

Neural networks have been applied to the speed estimation problem in many forms, either in combination with ZUPT [16], or standalone using classical architectures such as the LSTM. However, most often the networks use shallow architectures that cannot take advantage of the deep architectures, i.e., the ability to learn the most suitable high-level features. The pioneering work using deep architectures in this domain is [13], where the data are recorded using a smartphone on a large corpus of data. Therefore, it is sufficient to train a single feed-forward architecture. We have not collected such a large corpus for data with the mounted IMU data; hence, we use methods that are based on semi-supervised architectures. We show experimentally that autoencoder-based architectures outperform the feed-forward networks.

We have tested standard convolutional-recurrent architectures used in autoencoders [20,21] and designed a novel decoder using weighted harmonic signal. Our approach can be also seen as a generalization of weighting used in feature-based approaches [26], where the weighting strategy is fixed. In our approach, the weights as well as the latent space (i.e., features) are learned from the data.

### 2.3. Available Datasets

Existing datasets of IMU-style recordings were often designed for particular applications, such as patient control or daily life monitoring. Thus, they differ in the measurement setup, see survey [27]. We can recognize two principal types of datasets for motion speed estimation: (i) shoe-mounted IMUs, e.g., [27,28,29,30], and (ii) smartphone based data with varying positions (backpack or pocket) [14,31,32,33]. While the shoe mounted sensor is a traditional setup for feature-based methods, the deep-learning methods are predominantly trained on smartphone data in hand or pocket. Since the data do not contain information from both locations, it is impossible to select the most appropriate location. The dataset that we provide in this study is collecting data from three different locations of the sensors—shoe, shin, and thigh—to allow direct comparison of the sensor location for the same subjects under the same conditions, its relation to similar datasets is summarized in Table 2.

An essential piece of information for speed estimation is the measurement conditions and the speed reference. While a properly calibrated treadmill could be an excellent method to get the speed reference for stable speed, it is unnatural during speed transitions. Measuring the stable speed in discrete steps imposes the risk of an incomplete dataset. This is not an issue for feature-based methods with few parameters but becomes problematic for the deep learning methods that struggle to learn the speed between the discretization steps. Alternatives to obtain the speed reference are (i) optical systems such as VICON [30,34] which provides only a limited space for performing experiments which complicates especially the running scenarios, and (ii) visual SLAM [14,33] which does not suffer from space limitations but its accuracy of the ground-truth is low. Therefore, we used a different approach with the monowheel to obtain speed reference.

We provide both the data and code of our methods to guarantee reproducibility, which is still not standard in the field. Many datasets used in studies are only private, many papers do not contain detailed method description (SVM methods often use more than hundred features, but paper describes only a few), authors do not provide source codes, ground-truth is often missing or contaminated with error.

## 3. Methods

### 3.1. Data Acquisition: Sensors on a Single Leg

Cheap MEMS sensors BMX055 were used to obtain the IMU data measurement. Three locations—thigh, shin, and foot—were chosen strategically to capture good information about bipedal movement on a single leg. This setup is a compromise between the number of sensors and information gain. It is based on the underlying assumption that the motion of both legs is, to some extend, symmetric (this assumption is expected to hold for healthy subjects, such as firefighters).

**Figure 1:**
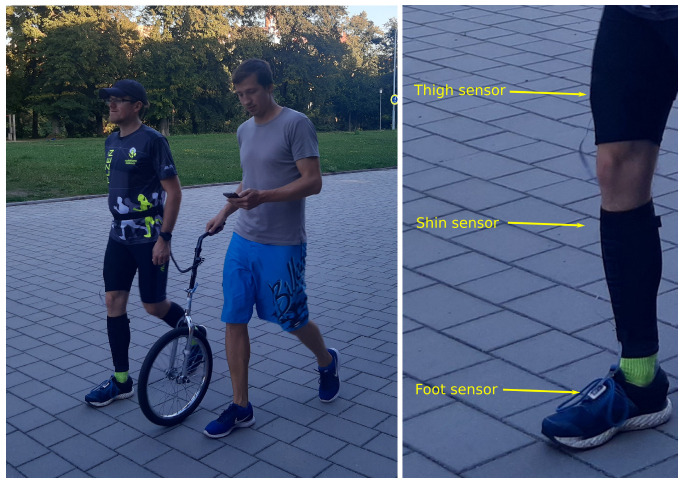
Illustration of the data acquisition procedure. (**Left**): collection of the IMU data and the reference mono-wheel data. (**Right**): details of the positioning of the IMU sensors on the leg.

The use of less accurate sensors poses another estimation challenge, and our results may thus differ from studies that used highly accurate and thus expensive sensors. For each location, one BMX055 was used to capture 6DOF information of an orthogonal accelerometer and gyroscope. The sensor breakboard was attached by adhesive to a 3D printed platform with elastic bands to obtain tight body attachment for thigh and shin placement. The foot sensor platform was attached via a hook-and-loop fastener to the shoe, see Figure 1 right. The data from sensors were fetched to ESP32 via I2C in speed mode. The data were redirected wirelessly from ESP32 over WiFi to an android phone which served as the data storage during the measurement. After some optimization, the final data rate fluctuates around 400 Hz.

The data were collected in an open environment with the reference speed collected using a custom modification of a monowheel. Actual measurement with the monowheel was obtained for all subjects by the same researcher to avoid data corruption of the subject’s natural movement; see Figure 1 left.

#### Collected Datasets

Eight subjects with various body proportions were measured, and their data are provided in the accompanying dataset (https://github.com/Josef4Sci/DeepGait/ accessed on 14 May 2022).

Data for each subject are stored in a single array, with each column containing data recorded in one time instant: $x_{t}$ organized as follows.

**Accelerometer** column 1:9, in three locations: 1:3 Thigh, 4:6 Shin, 7:9 Foot (conversion to m/s${}^{2}$ by multiplier 0.0024).
**Gyroscope** column 10:18 split into: 10:12 Thigh, 13:15 Shin, 16:18 Foot (conversion to deg/s by multiplier 0.061).
**Speed** column 19 (km/h).
**Time** column 20 (s).

**Figure 2:**
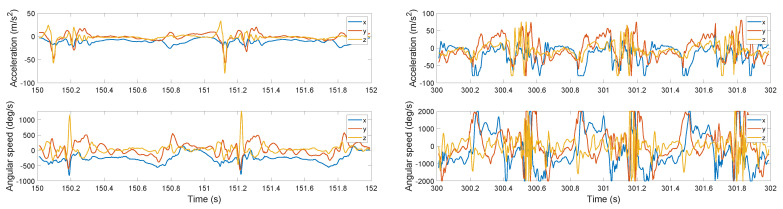
Example of the recorded IMU signals from the Foot IMU sensor for the first subject for walking speed of 6.5 km/h (**left**) and running speed of 24.2 km/h (**right**).

Examples of the recorded data are provided in Figure 2 for the first subject, where two short periods of walking and running are selected for comparison. Note that while in the walking phase, it is possible to identify the heel strike moments by a naked eye, it is much more demanding in the running phase. Moreover, the limits of the sensor, which are ±2000 deg/s gyroscope and ±80 $m/s^{2}$, are reached during the running phase. The saturation of the acceleration is critical for methods relying on its integration.

### 3.2. Problem Formulation

We are concerned with estimating an instantaneous speed of human bi-pedal motion using data from IMUs attached to the human body. The instantaneous speed at time index *t* is denoted $y_{t}$, and the instantaneous data record $x_{t}$. Due to insufficient information in a single observation, we predict the speed from a window $X_{t}=\left[x_{t-h},\ldots x_{t+h}\right]$ where *h* is a half-length of the window.

(1) $${\hat{y}}_{t}=f_{\theta }\left(X_{t}\right).$$

Here, $f_{\theta }\left(\right)$ is a parametric predictor function, with parameters θ (aggregating e.g., parameters of all layers of the neural network). We aim to approach the problem by supervised learning, i.e., recording dataset on multiple subjects i=1,…,N of both the speed $Y_{i}=\left[y_{i,1},\ldots ,y_{i,T_{i}}\right]$ and the IMU data $X=[x_{i,1},\ldots ,x_{i,T_{i}}]$ where $T_{i}$ is the length of the recorded time series. These data are used to train the predictor by minimizing a loss function between the predicted and the measured data.

(2) $$\begin{array}{cc} \hat{\theta } & =\arg\min_{\theta }L \end{array}$$

(3) $$\begin{array}{cc} L & =\sum_{i\in I_{\mathrm{train}}}\sum_{t=h+1}^{T_{i}-h}\mathrm{loss}\left(y_{t,i},X_{t,i}\right). \end{array}$$

The deep learning methods vary in the architecture of the neural network, yielding different types of parameters, and in the formulation of the loss function (3). These details will be elaborated in Section 3.3.

Note that various classical methods also fit into this formulation, however, the parametric space is much more restricted to contain e.g., tuning parameters of the data processing. Thus, we may consider the conventional feature-based methods as predictors with restricted degrees of freedom, while the deep-learning methods are free to learn the model structure from the data. The key challenge of the deep methods is to discover the right level of generalization, i.e., avoiding overfitting the training data with poor performance on the unseen (testing) data.

### 3.3. Deep Learning Methods

Even deep learning predictors are essentially just more complicated non-linear regression as defined in Section 3.1. Different model structures are represented by different architectures of the used network. The challenge is to find architecture that best corresponds with the nature of the studied problem. In this Section, we briefly review architectures that will be tested on the collected data. Specifically, we will compare two types of network architectures: (i) feed-forward architectures, and (ii) the semi-supervised variational autoencoder architecture. The former is a direct prediction method [10]. The latter is an extension of the feed-forward architecture by incorporating a generative model [35] as a tool for improving the generalization capabilities of the model.

#### Feed-Forward Networks

The feed-forward approach is a straightforward application of the neural network to the regression problem by defining a single network $f_{\theta }\left(X_{t}\right)$ and a mean square error loss.

(4) $$\mathrm{loss}\left(y_{t},X_{t}\right)=\left|\right|y_{t}-f_{\theta }\left(X_{t}\right){\left|\right|}_{2}^{2}.$$

The loss function is a classical least-squares approach with well-known solution for the linear model. A vast amount of neural architecture has been proposed and tested. For example, simple classical LSTM architecture has been tested on the speed estimation problem in [16]. Recent progress in deep learning indicates that much better results can be obtained by convolutional architectures. We will test the two most recent methods of this category:

**InceptionTime** is an architecture defined using convolutional layers with a bottleneck [18].
**Perceiver** is a complex architecture based on the attention mechanism [19].

These architectures have a number of hyper-parameters, mostly sizes, and dimensions of the latent variables, a detailed discussion of which is beyond the scope of this paper, see original publications for details. We have used reference implementation of these methods provided by their authors. The tuning of their hyper-parameters was performed using a uniform random search with ranges given in Table 3. The ranges were adjusted to ensure that the best results were not obtained at the border of the range.

### 3.4. Semi-Supervised Variational Autoencoders

While the feed-forward approach is methodologically simple, it often suffers from overfitting, i.e., achieving a good fit on the training data but poor on the test. While various regularization techniques were proposed, they are often hard to tune for good performance. An interesting approach to this problem is based on the use of autoencoders for regularization. Informally, the autoencoder projects the input information on the latent code—often of a much smaller dimension than the input—from which it tries to reconstruct the input as closely as possible. Specifically, the auto-encoder architecture defines two networks: (i) encoder generating the latent code $z_{t}=g_{\psi }\left(X_{t}\right),$ and (ii) decoder generating the input reconstructions of the input from the code $X_{t}^{\prime }=f_{\theta }\left(z_{t}\right)$. The training loss is the mean square error of the reconstruction:

(5) $${\mathrm{loss}}_{\mathrm{AE}}\left(y_{t},X_{t}\right)=\left|\right|X_{t}-X_{t}^{\prime }{\left|\right|}_{2}^{2}=\left|\right|X_{t}-f_{\theta }\left(g_{\psi }\left(X_{t}\right)\right){)||}_{2}^{2},$$

where the free variables are parameters of the neural networks ψ (encoder ) and θ (decoder). The autoencoder alone is too ambiguous to train and has to be coupled with a regularization. The most popular, and often the best performing in practice, is the Variational autoencoder [36]. The additional assumption is that the latent variable (code) is distributed as independent Gaussian $p\left(z_{t}\right)=N\left(0,I\right)$. The encoder does not provide an estimate of a single *z* but its distribution q(z|x) which is prescribed to have Gaussian form with mean $\mu_{\psi }\left(X_{t}\right)$ and standard deviation $\sigma_{\psi }\left(X_{t}\right)$, both functions being represented by neural networks. The loss of the autoencoder is computed as evidence lower bound, yielding the following formula.

(6) $${\mathrm{loss}}_{\mathrm{VAE}}\left(y_{t},X_{t}\right)=\left|\right|X_{t}-f\left(\mu \left(X_{t}\right)+\sigma \left(X_{t}\right)\circ \epsilon \right){\left|\right|}_{2}^{2}++\beta \mathrm{KL}(N\left(\mu \left(X_{t}\right),\sigma \left(X_{t}\right)\right)||N\left(0,I\right)).$$

The parameter β>0 governs compromise between the first (reconstruction) term and second (regularization) term of (6), KL(p||q) denotes the Kullback-Leibler divergence between probability distributions *p* and *q*, which is available in closed form for Gaussian distributions see [36] for details.

**Figure 3:**
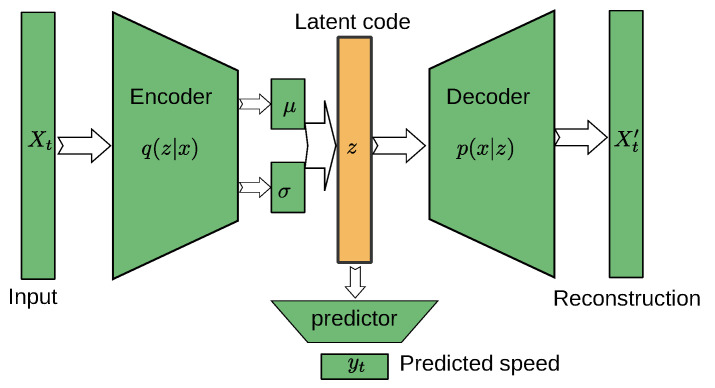
Schematic architecture of semi-supervised variational autoencoder. The path from the input $X_{t}$ to $y_{t}$ corresponds to the feed-forward path; the path from the input $X_{t}$ to prediction $X_{t}^{\prime }$ provides regularization.

The combination that we are using originates in semi-supervised learning that aims to combine the Variational Autoencoder with feed-forward predictors [35]. The goal is to use a simple weighted combination of the previous loss functions ((i) the prediction loss (4) and (ii) the VAE loss (6)) to yield the following:

(7) $$\begin{array}{c} \mathrm{loss}\left(y_{t},X_{t}\right)=\alpha ||y_{t}-h_{\theta }\left(\mu \left(X_{t}\right)+\sigma \left(X_{t}\right)\circ \epsilon \right){\left|\right|}_{2}^{2}+\left|\right|X_{t}-f\left(\mu \left(X_{t}\right)+\sigma \left(X_{t}\right)\circ \epsilon \right){\left|\right|}_{2}^{2}\\ +\beta \mathrm{KL}(N\left(\mu \left(X_{t}\right),\sigma \left(X_{t}\right)\right)||N\left(0,I\right)), \end{array}$$

where α is the weighting factor of the prediction quality. Note that by various choices of the weighting factors α and β we can recover various architecture. For example: (i) for β→0,α→0, (7) approaches pure autoencoder, and (ii) for α≫β,α≫1, it approaches the feed-forward predictor. Thus tuning optimal values of α and β allows obtaining the best of the combined approaches. Models of this type will be denoted as semi-supervised variational autoencoders (SVAE), and its architecture is illustrated in Figure 3. A wide range of such methods may be designed for various choices of the architecture of the involved neural networks.

Recent studies indicate that state-of-the-art performance on time series data is obtained by a combination of CNN layers (acting as adaptive filters) followed by LSTM layers capturing the dynamics of the process. While this architecture was not successful in the feed-forward setting, it worked very well as an encoder in VAE as proposed in [22]. However, motivated by the nature of our signal (see Figure 2), we investigated the possibility that the signal can be reconstructed using periodic functions. We, therefore, test two versions of the SVAE, differing in the decoder:

**Conventional Decoder:** SVAE-LSTM-CNN

With decoder being the reverse of the encoder, i.e., the LSTM layer followed by the deconvolution (ConvTranspose). This architecture is an extension of the classical LSTM autoencoder [22].

**Proposed Decoder:** SVAE-Sine

Motivated by the periodic nature of the generated signals, Figure 2, we propose the decoder in the form of weighted sine-waves:

$$X_{t}=W_{1}z_{t}\circ \sin\left(W_{2}z_{t}\tau +W_{3}z_{t}\right),\;\;\tau =t-h,\ldots ,t+h,$$

with learnable parameters $\theta =[W_{1},W_{2},W_{3}].$ This decoder has only one layer, and it thus much simpler than the LSTM-CNN decoder. We conjecture that this is the reason why SVAE-Sine was experimentally found to be more reliable than that of the LSTM-CNN version.

**Remark** **1.** *The architectures mentioned in this Section were selected from a larger pool of methods using preliminary studies. We have tested many simple architectures—such as plain LSTM autoencoers [16] or plain CNN networks—for a limited number of runs. However, the results of simple architecture were significantly worse than those of the above-described ones (e.g., it was tough to obtain a converging pure LSTM network). Since this agrees with previously reported experiments, e.g., [37,38], we removed these simple architectures from the study and performed the computationally expensive cross-validation study with Monte Carlo hyper-parameter search only on the four above-mentioned methods.*

Hyperparameters of the considered SVAE architectures are again sampled from ranges summarized in Table 4.

## 4. Experiments

### 4.1. Experimental Protocol

The evaluation procedure follows the leave-one-out cross-validation protocol [39]. Specifically, the methods were trained 8 times, with data collected on one subject being used as testing data, and the remaining data used for training and validation (85% for training, 15% validation). The validation data were used for monitoring convergence of the training procedure, which was stopped when the validation error did not improve for 20 consecutive steps. The training loss is the mean square error (4) or its augmented version (7).

If not stated otherwise, all reported accuracies are estimation errors of the *testing* subject, averaged over all 8 repetitions. The mean absolute error was chosen as the main evaluation metric:

(8) $$\mathrm{Err}=\frac{1}{8}\sum_{s=1}^{8}\frac{1}{t_{\max}}\sum_{t=1}^{t_{\max}}\left|y_{s,t}-f_{s}\left(X_{s,t}\right)\right|,$$

where $f_{s}\left(\right)$ is a model trained on data from all subjects except the *s*th. The error will be provided in the unit of the speed, i.e., km/h.

### 4.2. Conventional Feature-Based Methods

We will compare all tested deep-learning methods with state-of-the-art approaches based on heuristic/feature-based approaches. We have used the recent survey [24] for selecting the candidates. The methods are based on the integration of the speed from the acceleration sensor, using different features, such as detected heel-strikes for each limb, to calibrate the integration. Ten different variants of the features and their data processing are compared in [24], called method 1 to method 10, with the increasing complexity of the processing. The best performing method for their data was method 10 that uses information from the wrist sensor. Since we do not have sensors on the wrist, we compared only those not using their signals, i.e., methods in Table 5. The parameters of all relevant methods of [24]—i.e., the complementary filter cut frequency, the gyroscope scale error compensation parameter, and the accelerometer scale error compensation parameter—were optimized using Matlab’s fminsearch method for best accuracy (8) for each method independently.

The parameters obtained by optimization are not very intuitive, for example, the negative scaling for method 5. However, the error of speed estimation for more usual parameters was higher than that for the optimized. We conjecture that this is due to the nature of the collected signals (containing e.g., saturations, Figure 2) from a cheap and noisy sensor.

The best performance that was obtained on our data is Method 4 of [24], which uses the mid-stance to mid-stance segmentation to obtain the heel strike and toe-off indices. The estimation of yaw angle was manually set to zero constant because only the forward speed is measured.

### 4.3. Deep Learning Methods

For training and testing of all deep learning models, the data were resampled to isochronal 512 Hz and split into 2 s time windows with the reference speed set to the middle element of the window.

Since the number of the training samples is still low, the training data set for neural network training were augmented [40]. Two techniques were used to create the augmented samples: (i) sensor rotation, i.e., the measurements from one sensor were rotated by a small constant angle generated from Gaussian distribution with the standard deviation of 2.5°, and (ii) and a Gaussian white noise addition, using relative noise with standard deviation being 1% of the observed value. The sensor rotation simulates a slightly different sensor placement to clothes or body parts. A small portion of white noise was used to force the models to learn long-term dependencies.

### 4.4. Method Comparison for a Single Foot Sensor

For all four tested deep architectures—InceptionTime, Perceiver, SVAE-LSTM-CNN, SVAE-Sine—we have sampled 15 random draws from the ranges provided in Table 3 and Table 4, respectively. The leave-one-out cross-validation was performed for each hyper-parameter value, and the results were sorted according to their testing error (8). Tables with the three best hyperparameter values for the data using the single foot sensor are provided in the Appendix A. We note that the difference between the best hyperparameter values of individual methods is relatively small, indicating consistent performance with low sensitivity to individual hyper-parameter. Thus, we did not perform any averaging over the initial conditions of the network learning.

**Figure 4:**
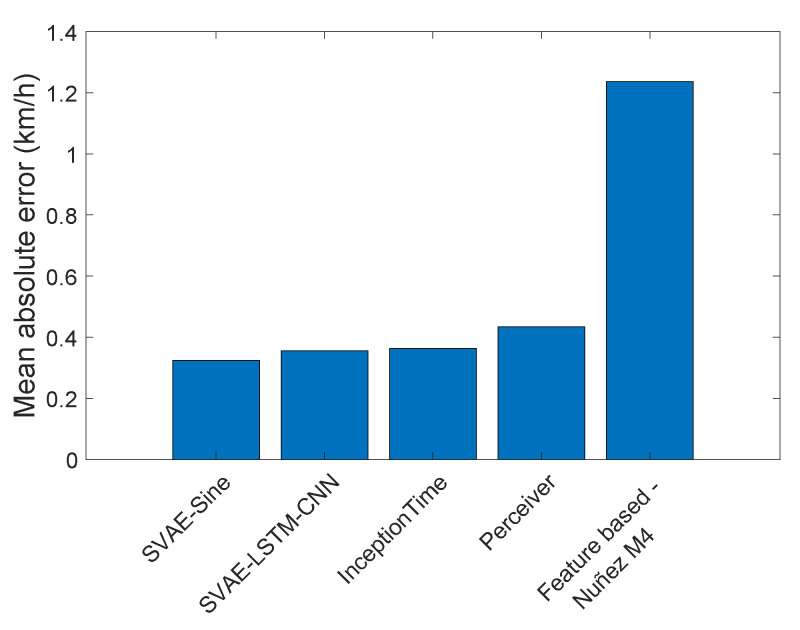
Mean absolute error of speed prediction obtained by the four tested deep methods and the best feature-based method [24] (M4 from Table 5). Results are averaged over 8 subjects of the leave-one-out cross-validation. All methods were optimized for best performance.

**Figure 5:**
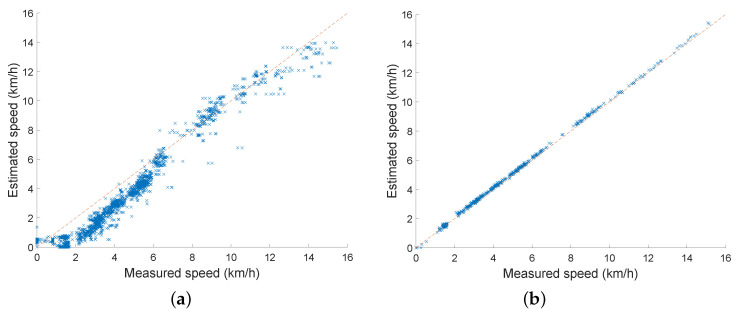
Scatter plot of measured and predicted speed of tested subject number 1 (unseen in training) for two prediction methods: the best performing conventional methods of [24] and the best performing deep method SVAE-Sine. (**a**) feature-based method 4 of [24]. (**b**) deep architecure SVAE-Sine. Dotted line indicates perfect reconstruction.

A summary of the speed estimation errors for all deep methods is displayed in Figure 4 in comparison with the best feature-based method. Note that all deep learning methods achieved significantly better results than the conventional feature-based methods. The difference between the approaches is even more striking when the measured and predicted speed on the tested subject (unseen in training) are displayed in the form of a scatterplot in Figure 5.

Note that the prediction of the deep method does not exhibit any significant distortions depending on the speed, which are visible in the results of the conventional method. Scatter plots for other subjects and also for other deep methods are similar to that for SVAE-Sine.

While the proposed SVAE-Sine model yields the lowest prediction error, we note that even feed-forward methods, such as the InceptionTime, provide comparable results. The limiting factor for improving the estimation is thus not the architecture of the network but more likely other issues such as (i) inter-subject variability, and (ii) informativeness of the sensor data.

### 4.5. Inter-Subject Variability

**Figure 6:**
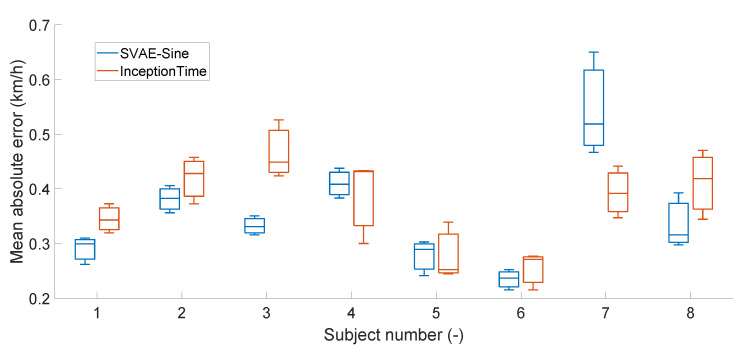
Boxplot of prediction error for all subjects in the study for the best performing SVAE-Sine architecture and the best feed-forward method, InceptionTime.

An illustration of the multi-subject variability is displayed in Figure 6 via a box plot of speed prediction errors for individual subjects in the cross-validation study, where the difference between subjects is clearly visible. The results of two methods are compared in this chart: SVAE-Sine as representative of the semi-supervised methods and InceptionTime as representative of the feed-forward networks. Both methods achieve consistent results with low error on some subjects, such as numbers 5 and 6. However, they differ on other subjects, such as numbers 3 and 7 where the winner is SVAE and InceptionTime, respectively. Differences between these subjects may be caused by insufficient training or other causes. We consider SVAE to provide better results due to a lower average error but also lower error on the majority of the subjects.

Note, however, that even the largest error in the most outlying subject number 7 is still significantly lower than errors of the conventional method (Figure 4).

### 4.6. Sensitivity to the Sensor Location

**Figure 7:**
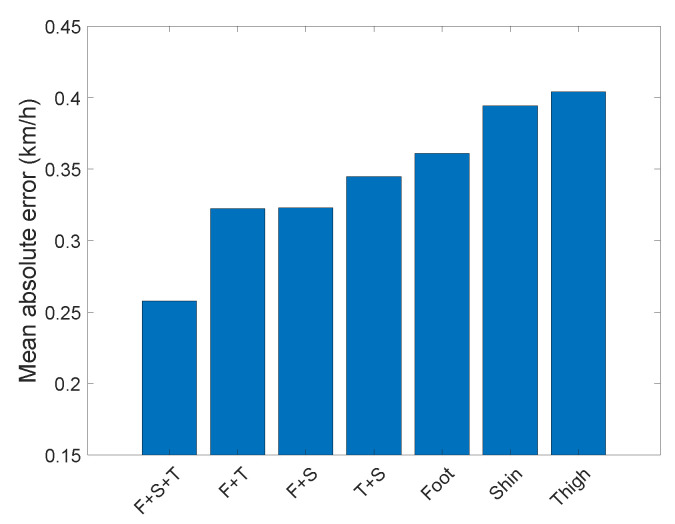
The average error of speed prediction of the SVAE-Sine method trained data from IMU sensors at different locations and their combinations.

In this Section, we investigate how much accuracy can be gained by using additional sensors. We will not investigate all architectures but focus only on the SVAE-Sine approach. The performance of the method was evaluated on all individual sensors, all couples, and all three sensors, see Figure 7. Hyperparameters of the architecture were those of the best performing architecture for the Foot sensor (Appendix A).

The foot seems to be the most valuable location for the sensor if it is used as a single sensor, as well as in tandem with a sensor in another location. The error of speed estimation monotonically decreases with the increasing number of sensors.

### 4.7. Additional Biometric Information

Biometric information such as the length of the leg or weight of the body may be relevant for the estimation of the motion speed from IMUs. The question for deep learning is whether the availability of such information improves prediction error. We have trained the SVAE-Sine model with two additional inputs: (i) the length of the leg, and (ii) the body mass of the subject. We found that in our study such an extension did not yield any significant improvement in the estimation error.

## 5. Discussion and Future Work

We have designed the dataset to measure the signal at three different positions that allows comparison of our results with both shoe-mounted and smartphone-based data. Previous studies indicate that since the smartphone is not coupled to the body movement properly (e.g., held in one hand), the estimates have higher error [33]. The situation where the smartphone is in the pocket corresponds to its placement on the thigh in our study. We have shown that it is the least accurate position of the all possible locations tested in our study (Figure 7), even though our error at this position is much lower than that from the smartphone (0.4 km/h for our method, 1.08 km/h for the smartphone [15]). Moreover, we have also shown that the error of the speed estimation is significantly reduced with an increasing number of sensors (0.25 km/h for three sensors). Fusion of multiple sensors is very straightforward in deep-learning methods, contrary to feature-based approaches. We conjecture that multiple sensors will be necessary to address more complicated motions such as knee-walking or ladder climbing. Due to the lowering prices of the sensors, we foresee a great potential for future research in the investigation of multi-sensor data e.g., integrated into special-purpose suits [41].

One of the key observations of our study is the inter-subject sensitivity of the speed estimates. We have shown in the cross-validation study that differences between subjects are much larger than between various deep-learning architectures (Figure 6). This highlights inherent difficulties in designing a universal speed estimation algorithm (which is the goal of all feature-based methods). On the other hand, it opens a way for personalization of the method. Benefit of algorithmic personalization of the estimation algorithms for each individual were already studied in [3], and deep learning is in a great position to address this need. We foresee a great potential for the deep learning technique of pre-training [42] which prepares universal representation on a larger data corpus at high computational cost and finalizes training for personalized models with a lower number of the data and lower computational cost.

## 6. Conclusions

Estimation of the speed of motion from IMU attached to a human body has been dominated by feature-based methods. We have collected a dataset of eight subjects with ground truth speed and provide them publicly as a benchmarking dataset. We have demonstrated that recently developed deep learning architectures are able to provide much closer estimates than conventional methods on this dataset. Moreover, we have proposed a modification of the existing semi-supervised variational auto-encoder using a decoder in the form of a dense layer with sinusoidal activation functions. The proposed deep architecture was tailored for this application and outperformed, on average, the general-purpose state-of-the-art methods on this task. Estimation error was always evaluated on the tested dataset (unseen during the training). The estimation error monotonically decreases with an increasing number of sensors on the body. The primary source of variability of the error is the human subject. However, even the subject with the largest error of the deep-learning method has a lower error than that of the best tested conventional feature-based method. On the other hand, the various architectures of deep networks seem to yield comparable performance. Therefore, we recommend focusing on the data acquisition and subject variability rather than details of network architectures for future work.

## Figures and Tables

**Table 1:** Summary of existing approaches using IMU for pedestrian dead reckoning and their key properties.

Method	Year	Public Data	Features	Neural Architecture	Run	Source Code	Placem. Study	Note
[13]	2019	Yes	–	CNN-LSTM	No	No	No	Smartphone
[9]	2020	No	ZUPT	–	No	No	No	
[1]	2017	No	Custom	–	No	No	No	
[7]	2019	No	ZUPT	–	Yes	No	–	Adaptive threshold
[6]	2017	No	ZUPT	–	Yes	No	–	Missing ground truth
[16]	2020	No	ZUPT+NN	LSTM	No	No	No	
[24]	2020	No	ZUPT	–	No	Yes	–	Multiple methods
[23]	2017	No	ZUPT	–	No	No	–	Kalman
[8]	2017	No	ZUPT	–	Yes	No	–	Adaptive threshold
[3]	2019	No	Custom	–	Yes	No	No	Wrist, Personalized
**ours**	-	Yes	–	CNN, RNN, VAE	Yes	Yes	Yes	

**Table 2:** Summary of existing datasets for human motion speed estimation.

Dataset	Year	Run	Sensor Location	Device	Reference	Available
[31]	2014	No	waist, shirt pocket, bag	Smartphone	Human label	Yes
[32]	2018	No	hand, pocket, bag, trolley	Smartphone	VICON	Yes
[34]	2016	Yes	ankles	VICON	Threadmill ^1^	Yes
[14]	2019	No	hand, pocket, bag	Smartphone	Visual SLAM	Yes
[33]	2018	No	hand, pocket, bag, body	Smartphone	Visual SLAM	Yes
[28]	2015	No	foot	IMUs	Human label	on demand
[29] ^2^	2014	No	foot	IMUs	Human label	on demand
[30]	2017	No	foot	IMUs	Optical system	Yes
**ours**	-	**Yes**	**foot, shin, thigh**	IMUs	Monowheel	Yes

^1^ Speed labels can be reconstructed from the available data. ^2^ Old patients.

**Table 3:** Hyper-parameter ranges of deep feed-forward architectures.

InceptionTime		Perceiver	
**Hyper-Parameter**	**Range**	**Hyper-Parameter**	**Range**
number of filters	[2, 4, 8, 16, 32]	Number of freq. bands	[6, 12]
kernel sizes	[[5, 11, 21], [11, 21, 41], [21, 41, 81]]	Maximum frequency	[3, 5, 10, 15]
		Depth	[6, 12]
bottleneck channels	[2, 4, 8]	Number of latents	[128, 256, 512]
		Dimension of latents	[64, 128, 256]
		Dimension of cross layer	[512, 256, 128]
		Dim. of att. head for cross layer	[64, 32, 16]
		Dim. of att. head for latents	[64, 32, 16]

**Table 4:** Hyper-parameters of the semisupervised VAE approach.

Encoder		Decoder	
**Hyper-Parameter**	**Range**	**Hyper-Parameter**	**Range**
Convolution channels	[1, 2, 4, 8, 16]	Sine: size of hidden layer	[10, 50, 100]
Size of hidden layer	[128, 256, 512]	LSTM-CNN: same as encoder	
Depth of hidden layer	[1, 2]		
Length of latent *z*	[64, 128, 256]		
Predictor weight α	[0.1, 0.01, 0.001, 0.0001]		
KL weight β	[1$\times 10^{-4}$, 1$\times 10^{-5}$, 1$\times 10^{-6}$, 1$\times 10^{-7}$]		

**Table 5:** Tuning parameters and performance of the conventional methods from [24] using their numbering.

ID	Method Features	Scale 1	Scale 2	Cut-Off Freq.	Error [km/h]
M2	heel-strike to heel-strike segmentation	1.0	1.2	0.82	4.9
M4	mid-stance to mid-stance segmentation	0.6	5.6	0.98	1.2
M5	M4 + gravity compensation	3.2	−3.0	0.80	3.7
M7	mid-swing to mid-swing segmentation	−0.02	1.9	$7\times 10^{-18}$	18.2
M8	M7 + outlier elimination	−0.002	1.9	$5\times 10^{-17}$	10.8

## Data Availability

All data collected during this study and all code to reproduce our experiments is available from: https://github.com/Josef4Sci/DeepGait/, accessed on 14 May 2022.

## Appendix A. Details of Hyperparameter Selection

In this section, we provide a review of the top-performing hyperparameters selected for each of the tested deep architectures. The hyperparameters are sorted according to the mean testing error in decreasing order. Note that the architecture’s performance is quite consistent, and differences between hyperparameters settings are often smaller than differences between the methods. The tables below show the top three errors with hyperparameter settings from 15 draws for each architecture.

**Table A1:** Summary of the best hyper-parameters of the Autoencoder: CONV-LSTM-CONV.

Conv_Channels	Hidden_Size	Hidden_Layer_Depth	Latent_Length	α	β	Error km/h
8	128	1	64	0.1	$1\times 10^{-7}$	0.3552
8	128	1	128	0.01	$1\times 10^{-5}$	0.3814
8	128	1	64	0.01	0.0001	0.3844

**Table A2:** Summary of the best hyper-parameters of the Autoencoder: CONV-LSTM-SineNet.

Conv_Channels	Hidden_Size	Hidden_Layer_Depth	Latent_Length	α	β	Sin_Depth	Error km/h
16	256	1	128	0.01	$1\times 10^{-7}$	100	0.3238
8	256	1	128	0.01	$1\times 10^{-7}$	50	0.3640
8	256	1	64	0.1	$1\times 10^{-4}$	10	0.3693

**Table A3:** Summary of the best hyper-parameters of the InceptionTime.

n_Filters	Kernel_Sizes	Bottleneck_Channels	Error km/h
16	[21, 41, 81]	8	0.3630
16	[11, 21, 41]	8	0.3662
8	[21, 41, 81]	4	0.3799

**Table A4:** Summary of the best hyper-parameters of the Perceiver.

Num_Freq_ Bands	Max_ Freq	Depth	Num_ Latents	Latent_ Dim	Cross_ Dim	Cross_ Dim_Head	Latent_ Dim_Head	Error km/h
6	10.0	6	256	128	256	32	64	0.4339
6	15.0	6	256	128	512	32	16	0.4691
12	15.0	12	512	256	128	64	64	0.4956
